# Eugenol Activity Against *Escherichia coli* and *Staphylococcus aureus*: Biofilm Disruption and Additive Interactions with Antibiotics

**DOI:** 10.1007/s00284-026-05201-9

**Published:** 2026-10-23

**Authors:** Victor Alves Carneiro, Águida Maria Albuquerque Azevedo, Benise Ferreira da Silva, Ramaiana Soares Melo, Antônio Mateus Gomes Pereira, Rafaela Mesquita Bastos Cavalcante, Francisco Eduardo Aragão Catunda Junior

**Affiliations:** 1 Laboratory of Biofilms and Antimicrobial Agents (LaBAM/NUBEM), INTA University Center – UNINTA, 62.050-100, Sobral, Ceará Brazil; 2 Faculty of Medicine, INTA University Center (UNINTA), Vilebaldo Aguiar 133, Fortaleza, Ceará 60192-010 Brazil; 3 Northeast Network of Biotechnology Program (RENORBIO), State University of Ceará, Campus Itaperi, Fortaleza, 60714-903 Brazil; 4 Center for Exact, Natural and Technological Sciences, State University of the Tocantina Region of Maranhão, Imperatriz, 65901-480 Brazil

## Abstract

The emergence of multidrug-resistant bacteria and their ability to form biofilms represent significant challenges in infection control. This study evaluated the antibacterial and antibiofilm activities of eugenol (Eug) against *Escherichia coli* and *Staphylococcus aureus* from food and clinical sources. Antibacterial activity was assessed using disk diffusion, broth microdilution (MIC/MBC), and bacterial growth kinetics. Antibiofilm activity was measured by crystal violet staining, CFU enumeration, and scanning electron microscopy (SEM). Checkerboard assays evaluated the combinatorial effect of Eug with ciprofloxacin and oxacillin. Eug exhibited inhibition zones of 13–22 mm and MIC/MBC values of 1000–2000 µg/mL. Subinhibitory concentrations significantly delayed bacterial growth. Biofilm biomass was reduced by up to 90% in *E. coli* and 80% in *S. aureus*, with SEM confirming structural alterations. Checkerboard assays revealed additive interactions with antibiotics. These findings provide additional evidence of Eug’s antibacterial properties and support further investigation into its combinatorial effects with antibiotics against biofilm-associated challenges.

## Introduction

Excessive and often inappropriate antibiotic use, combined with bacterial adaptive mechanisms, has significantly contributed to the global rise of multidrug-resistant (MDR) bacterial infections. According to the World Health Organization (WHO), antimicrobial resistance directly caused more than 1.27 million deaths in 2019, with nearly 5 million deaths globally [1, 2]. These infections result in prolonged hospitalizations, increased use of high-cost antibiotics, and higher mortality rates, further underscoring the urgent need for novel antimicrobial alternatives [3–5].

Infections caused by resistant bacteria are not confined to hospital environments and represent a growing threat in non-clinical settings, particularly in the food industry. Contaminated food-contact surfaces can serve as primary reservoirs for pathogenic strains, promoting cross-contamination and spreading resistant strains throughout the production and supply chain [6, 7]. Additionally, the bacterial ability to form biofilms, structured communities of microbial cells embedded in a self-produced extracellular matrix, contributes to persistent contamination [8]. This matrix consists mainly of polysaccharides, proteins, extracellular DNA, and water and serves as a physical and biochemical barrier that impairs antimicrobial penetration and enhances bacterial tolerance [9]. Among biofilm-forming pathogens, *Escherichia coli* and *Staphylococcus aureus* represent major Gram-negative and Gram-positive species associated with food contamination and device-associated infections [10, 11]. Moreover, these organisms are frequently recovered from mono- and polymicrobial biofilms and exhibit a remarkable capacity to persist under adverse environmental conditions [12]. In addition, phenotypically distinct survival subpopulations/states can be found, such as persisters, small colony variants, and viable but non-culturable (VBNC) cells [13–15].

This scenario has intensified the search for alternative therapeutic strategies, including natural compounds with antimicrobial properties. Phytochemicals derived from essential oils, plant extracts, and secondary metabolites have gained significant attention for their ability to interfere with bacterial growth, quorum sensing, and biofilm architecture [4, 16]. These compounds have attracted attention as potential complementary strategies to conventional antimicrobials, particularly in the context of increasing bacterial resistance and biofilm-associated infections [7, 17].

Among them, eugenol (Eug), a phenolic constituent primarily found in clove (*Syzygium aromaticum*) essential oil, stands out for its broad-spectrum pharmacological properties [18]. In addition to its well-established antimicrobial, anti-inflammatory, and analgesic effects, Eug has demonstrated potential anticancer and antioxidant activities, which have been explored in both medical and pharmaceutical contexts [19, 20]. Recent studies have highlighted its ability to inhibit pathogenic bacterial growth, including by disrupting biofilms, making it a valuable candidate for alternative or adjunct antimicrobial therapies [21, 22]. Although studies have documented Eug’s antimicrobial properties, fewer have examined its effects on mature biofilms and its combinatorial interactions with conventional antibiotics against resistant Gram-positive and Gram-negative bacteria. Therefore, this study aimed to assess Eug’s antibacterial and antibiofilm activities and its interactions with conventional antibiotics against *E. coli* and *S. aureus* isolates.

## Materials and Methods

### Antimicrobial Substances

Eugenol (Farmos LTDA, Engenho Novo, RJ, Brazil) was prepared at 16 mg/mL in sterile Brain Heart Infusion (BHI, HiMedia, Mumbai, India) broth supplemented with 1% Tween 80. The conventional antibacterial agents, oxacillin (Oxa; 500 mg, Blau Pharmaceutical SA, São Paulo, Brazil) and ciprofloxacin (Cip; 2 mg/mL, Fresenius Kabi, São Paulo, Brazil) were diluted in 0.9% sterile saline to the required concentrations for each assay.

### Microorganisms and Culture Conditions

The antibacterial activity of eugenol was evaluated against six bacterial isolates, including three *E. coli* strains (P12, P25, and P36), previously isolated from fresh food by Almeida et al. (2017) and three *S. aureus* strains (2B, 5B, and 7B), isolated from hospital patients by Melo et al. (2019) [23, 24]. In addition, standard reference strains *E. coli* ATCC 11303 and *S. aureus* ATCC 6538 were included. Table 1 details the resistance profiles of all strains, previously determined by the authors.

**Table 1:** Bacterial strains and their respective sources, as well as antibiotic resistance profiles previously characterized by Almeida et al. (2017) [22] and Melo (2019) [23]

Species	Code	Source	Antibiotic resistance	Classification
*E. coli*	11,303	ATCC	None	Sensitive
	P12	Fillet fish	AMP, CFL, CIP, NOR, NAL, AMC (I)	MDR
	P25	Fillet fish	AMP, CFL (I), AMC (I)	Non-MDR
	P36	Fillet fish	AMP, CFL (I), AMC (I)	Non-MDR
*S. aureus*	6538	ATCC	None	Sensitive
	2B	Soft tissues	ERT, CLIN, BZP	Non-MDR
	5B	Human blood	ERT, CLIN, CIP, NOR, MOX, BZP, OXA, RIP (I)	MDR
	7B	Human blood	BZP, OXA	Non-MDR

Sensitive: susceptible to all antibiotics tested; AMC: Amoxicillin/Clavulanic Acid; AMP: ampicillin; BZP: benzylpenicillin; CFL: Cefelotin CIP: ciprofloxacin; CLIN: clindamycin; ERT: erythromycin; MOX: Moxifloxacin; NAL: nalidixic acid; NOR: norfloxacin; OXA: oxacillin; RIP: Rifampicina (I): intermediate resistance

Initially, bacterial cells were recovered from frozen stock cultures maintained at −80 °C and inoculated into 5 mL of Brain Heart Infusion (BHI) broth, then incubated at 37 °C overnight. Subsequently, 50 μL were transferred into fresh BHI broth (1:10 dilution) and incubated under the same conditions until the late exponential growth phase was reached. Before each assay, the bacterial suspensions were adjusted to a turbidity equivalent to the 0.5 McFarland standard (~ 10^8^ CFU/mL) and further diluted in fresh BHI to obtain the appropriate cell concentration for each experimental procedure.

### Disc-Diffusion Method

The antibacterial activity of Eug was first determined by the disk diffusion method [24]. Briefly, a sterile embedded cotton swab was inserted into a bacterial suspension adjusted to 10^8^ CFU/mL in 0.85% NaCl, rotated against the tube wall, and spread across the surface of Mueller–Hinton agar (MHA) plates (Acumedia®, MI, USA). Then, three 6 mm diameter disks (Laborclin®, Pinhais, Brazil) impregnated with 10 μL of pure Eug were placed equidistantly on the MHA surface. After 24 h of incubation at 37 °C, the antibacterial effect was evaluated by measuring the diameter of the inhibitory zones (IZD) in millimeters, and the results were expressed as the mean of three different determinations.

### Microdilution Test

The minimum inhibitory concentration (MIC) was determined using a standard microdilution test in 96-well polystyrene plates (KASVI, Paraná, Brazil), adapted from CLSI M07-A10 guidelines with modifications [25]. Two-fold serial dilutions were prepared in BHI medium to obtain 100 μL of Eug at concentrations ranging from 500 to 16,000 μg/mL. Subsequently, 100 μL of a bacterial suspension adjusted to 10^6^ CFU/mL in fresh BHI was added to each well, resulting in 5 × 10^5^ CFU/mL and halving the Eug concentration. The plates were incubated at 37 °C for 24 h, and MIC was determined as the lowest compound concentration that visually inhibited bacterial growth.

Additionally, bacterial growth kinetics were monitored during incubation by measuring absorbance at 620 nm hourly for 18 h (*E. coli*) and 24 h (*S. aureus*) under untreated, ½ MIC, and MIC treatment conditions. The extent of growth was quantified by calculating the area under the curve (AUC) for each treatment condition. To determine the minimum bactericidal concentration (MBC), 10 μL aliquots from wells exhibiting no visible bacterial growth were inoculated onto BHI agar plates and incubated at 37 °C for 24 h. Following incubation, the MBC was defined as the lowest concentration of the compounds that reduced ≥99.9% of the initial inoculum on the agar surface. Fresh BHI supplemented with the corresponding Tween 80 was used as a negative control.

### Antibiofilm Activity

Preformed 24 h biofilms were obtained by incubating 10^6^ CFU/mL bacterial inoculum in BHI at 37 °C in 96-well flat-bottom plates. After washing to remove planktonic cells, the biofilms were treated with eugenol at concentrations ranging from 8,000 to 1,000 μg/mL for 4 h at 37 °C. Untreated biofilms were incubated with fresh BHI/Tween only as negative controls. Residual biofilm biomass was quantified by crystal violet (CV) staining, following methanol fixation, staining with 0.1% CV solution, washing, and solubilization with 33% glacial acetic acid. Absorbance was measured at 590 nm to estimate biomass [26]. For viable cell enumeration, biofilms in separate plates were washed, treated with 0.85% NaCl, and sonicated for 5 min to detach cells. Serial 1:10 dilutions were plated onto BHI agar and incubated at 37 °C for 24 h to determine CFU/mL [21].

### Scanning Electron Microscopy

The structural changes in *E. coli* (P12) and *S. aureus* (5B) biofilms induced by MIC-based Eug treatment were evaluated using scanning electron microscopy (SEM – Inspect S50 – FEI Company, Oregon, USA) [21]. The biofilm was formed from a cell suspension (10^6^ CFU/mL) in fresh BHI for 24 h at 37 °C in 24-well plates containing glass slides (1 cm^2^). The slides were then washed three times with 0.85% NaCl, and the cells adsorbed on the surface were treated with Eug at MIC values for 4 h at 37 °C. For the untreated group, we used fresh BHI/Tween 80. Before SEM analysis, cells were pre-fixed with 2% glutaraldehyde for 1 h, dehydrated in increasing ethanol concentrations (10, 30, 50, 70, 90, and 100%) for 20 min each, and dried at room temperature. Slides were then placed on carbon tape on the aluminum sample Holder, coated with gold (Emitech Q150T, Lewes, UK), and imaged by SEM at 20 kV.

### Checkerboard Assay

The combinatorial effect of Eug with Cip against Cip-resistant *E. coli* (P12) and with Oxa against Oxa-resistant *S. aureus* (5B) was evaluated using the checkerboard assay, following White et al. (1996), with minor adaptations [27]. These antibiotics were selected based on the previously characterized resistance profiles of the strains to investigate whether eugenol could modulate the activity of clinically relevant conventional antibiotics against resistant bacteria.

Two-fold serial dilutions of each antimicrobial agent were prepared separately in BHI medium using 96-well microtiter plates. Briefly, 50 μL of Eug was added in rows at increasing concentrations, while the antibiotics were distributed in columns at serial dilutions based on their individual MICs. The contents of both plates were then combined to yield unique concentration combinations. Next, 100 μL of bacterial inoculum (10^6 CFU/mL) was added to each well, and the plates were incubated at 37 °C for 24 h. The fractional inhibitory concentration index (FICi) was calculated as FIC_A_ + FIC_B_, where FIC_A_ corresponds to the MIC of compound A in combination divided by the MIC of compound A alone, and FIC_B_ corresponds to the MIC of compound B in combination divided by the MIC of compound B alone. Interactions were interpreted according to EUCAST (2000), as synergistic (FICi ≤ 0.5), additive (FICi > 0.5 to ≤ 1), indifferent (FICi > 1 to < 2), or antagonistic (FICi ≥ 2) [28].

### Statistical Analysis

All experiments were performed in three independent assays. Bacterial growth level (growth kinetics) was expressed as mean ± SD by AUC calculated for each experimental condition. AUC values, biofilm biomass, and viable cell count (CFU/mL) were analyzed using one-way ANOVA followed by Tukey’s multiple-comparison test in GraphPad Prism 8.4.3 (GraphPad Software, Inc., San Diego, CA, USA). Differences were considered statistically significant at p < 0.01.

## Results

### Antibacterial Effect

Eugenol exhibited IZD values ranging from 13 to 15 mm for *E. coli* and 15 to 22 mm for *S. aureus*. Notably, the MIC and MBC for all *E. coli* were 2,000 µg/mL, while for *S. aureus* were 1,000 µg/mL, indicating that Eug is bactericidal, with MBC/MIC ratio of 1 for all tested microorganisms. Table 2 shows the IZD, MIC, MBC, and MBC/MIC for Eug against the tested bacteria.

**Table 2:** Antimicrobial activity of eugenol against isolates of *Escherichia coli* and *Staphylococcus aureus*, by inhibition zone diameter (IZD), minimum inhibitory concentration (MIC), and minimum bactericidal concentration (MBC), along with the MBC/MIC ratio for each strain. ATCC: American Type Culture Collection

Bacterial strains	Code	IDZ (mm)	MIC (µg/mL)	MBC (µg/mL)	MBC/MIC
*E. coli*	ATCC 11303	14	2,000	2,000	1
	P12	15			
	P25	13			
	P36	15			
*S. aureus*	ATCC 6538	18	1,000	1,000	1
	2B	16			
	5B	22			
	7B	15			

**Fig. 1:**
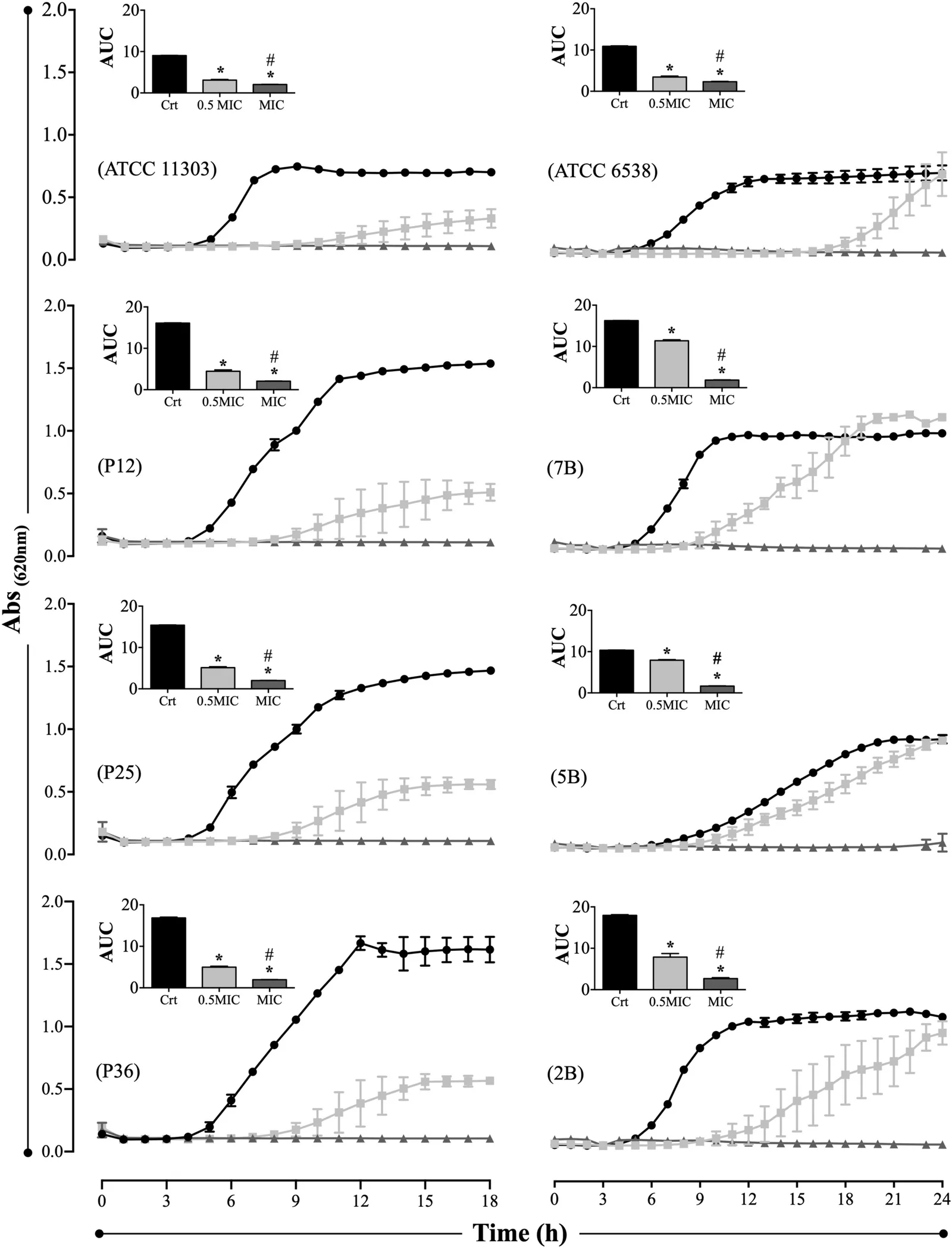
Growth curve (lines) of *Escherichia coli* (left side) and *Staphylococcus aureus* (right side) under ½ MIC (light gray) and MIC (dark gray) eugenol amounts over 18 and 24 h of incubation. Bacterial growth was quantified by the area under the curve (AUC, bars). For the untreated group (black), only BHI + Tween 80 was used. Statistically different from the control group (*) and eugenol amounts (#) by one-way ANOVA followed by Tukey’s multiple-comparison test (p < 0.01)

Growth curve analyses further confirmed its antibacterial effect, showing that even at sub-MIC (½ MIC), it significantly delayed bacterial proliferation by prolonging the lag phase and reducing exponential growth. Eug treatment (½ MIC) in all *E. coli* strains resulted in lower optical density values than the untreated control. For *S. aureus*, although an extended lag phase was also observed, the bacteria eventually resumed growth, reaching absorbance levels comparable to the control group. In contrast, no bacterial growth was observed at MIC concentrations for either species. Figure 1 illustrates the impact of Eug on the growth kinetics of *E. coli* and *S. aureus* strains at ½ MIC and 1 × MIC.

### Antibiofilm Activity

**Fig. 2:**
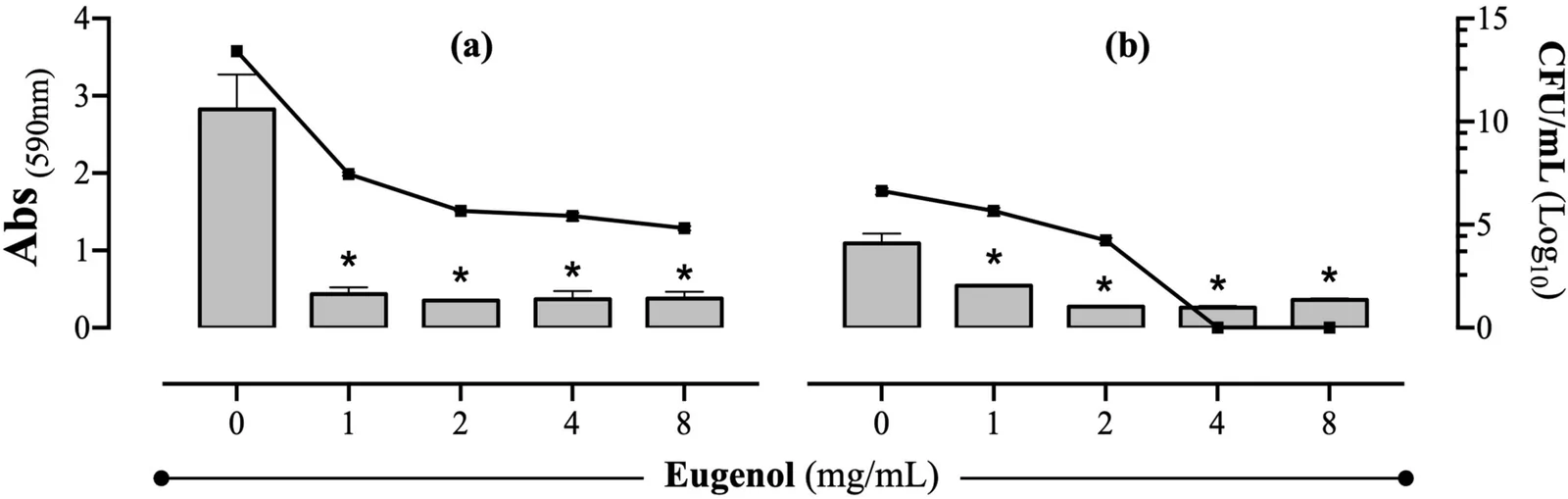
Antibiofilm activity of crescent concentrations of eugenol against preformed biofilm of *E. coli* P12 (**a**) and *S. aureus* 5B (**b**). Biomass quantification by crystal violet staining (OD 590 nm, bars) and cell enumeration by colony count (log_10_ CFU/mL, lines). * Significantly different from the control group by one-way ANOVA with Tukey’s test (p < 0.01)

Eug exhibited pronounced activity against preformed biofilms of *E. coli* (P12) and *S. aureus* (5B), reducing biofilm biomass by approximately 90% and 80%, respectively, compared with the untreated group. Treatment significantly reduced CFU counts in both strains (Fig. 2). Notably, while biomass reduction appeared to be dose-independent, the decrease (> 3 log_10_) in viable cell counts reinforced the effect of increasing Eug concentrations on biofilm-embedded bacteria.

### Scanning Electron Microscopy

**Fig. 3:**
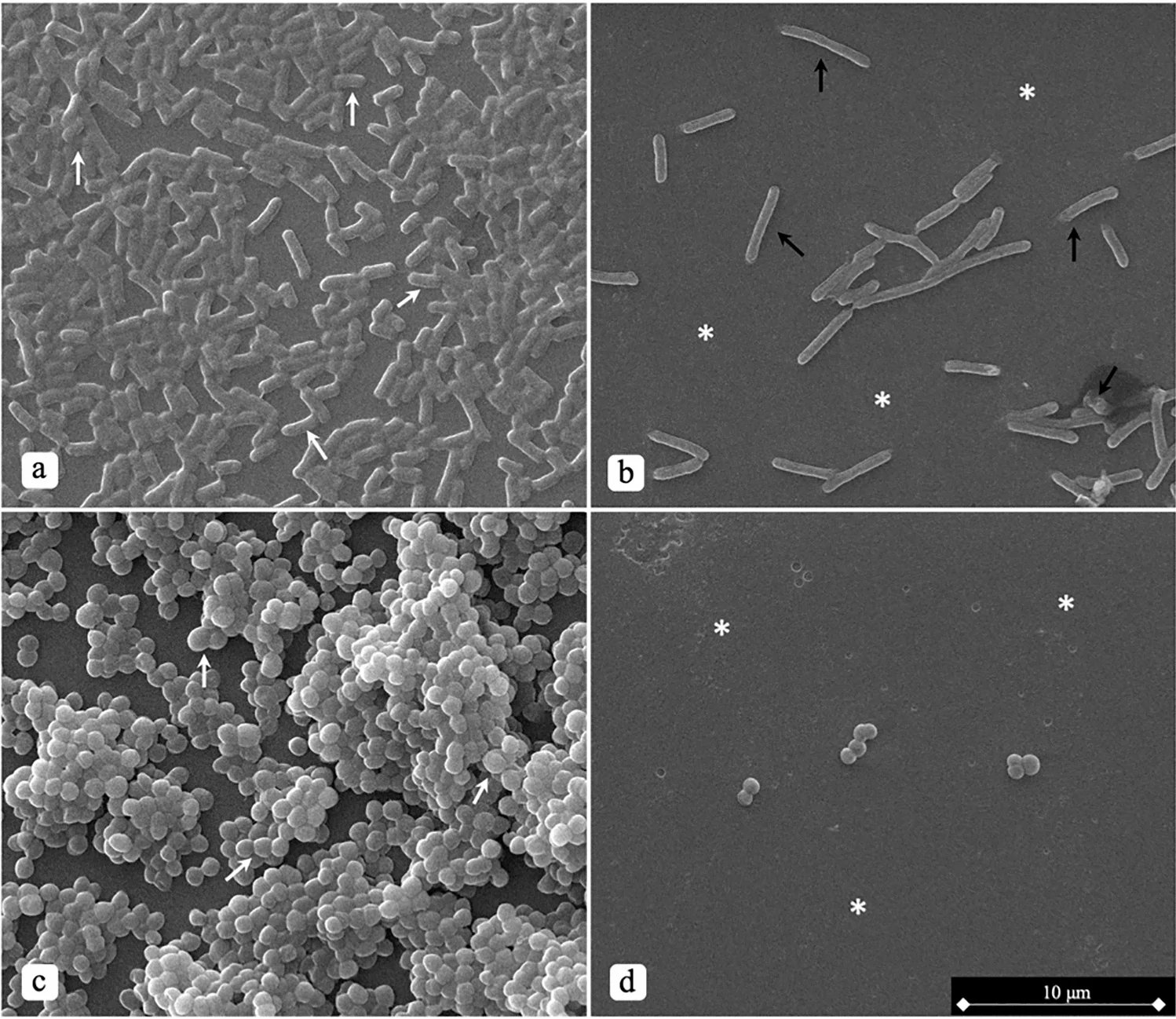
Scanning electron microscopy (SEM) images showing the eugenol impact on preformed 24 h-biofilms of *Escherichia coli* (P12) and *Staphylococcus aureus* (5B). Untreated (**a**) and Eug-treated (**b**) *E. coli* biofilm. Untreated (**c**) and Eug-treated (d) *S. aureus* biofilm. Intact rod-shaped and coccus-shaped cells: white arrows. Elongated bacterial cells: black arrows. Exposed surface areas: white asterisks

SEM images showed marked morphological differences between untreated and Eug-treated biofilms. Untreated *E. coli* biofilm showed a dense, well-organized community of intact rod-shaped cells (white arrows), with extensive surface aggregation characteristic of biofilm architecture (Fig. 3a). In contrast, *E. coli* biofilms treated with Eug at 1 × MIC showed reduced biofilm density (Fig. 3b). The remaining elongated or damaged bacterial cells (black arrows) appeared sparsely distributed, with large areas of the surface exposed (white asterisks).

A similar pattern was observed for *S. aureus* biofilms. The control displayed a thick, multilayered biofilm of tightly clustered coccoid cells with robust intercellular adhesion (Fig. 3c). However, following Eug treatment at 1 × MIC, a reduction in cell density was evident (Fig. 3d). Only a few individual cells remained adhered, and most of the biofilm structure was absent. SEM images show qualitative morphological disruption but do not indicate complete biofilm eradication, mainly at 1 × MIC.

### Combinatorial Effect

The strains *E. coli* (P12) and *S. aureus* (5B) were selected based on their resistance to Cip and Oxa, respectively. Cip was used for *E. coli,* while Oxa was used for *S. aureus*. The study demonstrated that Eug/Cip and Eug/Oxa combinations showed an additive effect, with FICi ranging from 0.75 to 1.0. In *E. coli* P12, the Eug/Cip combination reduced the MIC of Eug by 4-fold and Cip by 2-fold, while in *S. aureus* 5B, the Eug/Oxa combination reduced both MICs by 2-fold. No antagonistic combinations (FICi ≥ 2) were observed. All inhibitory concentration values ​​of individual and combined antimicrobial substances, FICi values​​, and x-fold reduction of antimicrobial substances for each strain are shown in Table 3.

**Table 3:** Fractional inhibitory concentrations index (FIC*i*) of ciprofloxacin (Cip) and eugenol (Eug) against CIP-resistant *Escherichia coli* (P12) and oxacillin (Oxa) combined with Eug against Oxa-resistant *Staphylococcus aureus* (5B). Add: additive

Bacterial strains	Antimicrobial agents	MIC (µg/mL)		FIC*i*	x-fold reduction
		Individual	Combination		
**P12**	Eug	2,000	500	0.75 (Add)	4×
	Cip	62.50	31.25		2×
**5B**	Eug	1,000	500	1.00 (Add)	2×
	Oxa	2,000	1,000		2×

## Discussion

The antibacterial activity observed is consistent with previous reports demonstrating its efficacy against Gram-negative and Gram-positive bacteria. Eug showed IZD 13–22 mm and antibacterial effects between 1,000 and 2,000 µg/mL (Table 2), supporting previous findings that classify it as bactericidal at high concentrations [29–31]. Similar concentrations have been reported for phenolic phytochemicals, whose antibacterial activity is often attributed to nonspecific membrane interactions rather than to highly selective intracellular targets [32].

The AUC reduction observed at sub-MIC indicates that Eug can alter bacterial growth dynamics even below its inhibitory concentration (Fig. 1). However, sub-MIC levels may exert distinct physiological effects on bacteria. Recent studies show that sub-MIC exposure can stimulate biofilm formation, particularly in *S. aureus*, depending on antimicrobial concentration and environmental conditions [33].

The antibiofilm activity of Eug aligns with that of Yadav et al. (2015), who reported that Eug effectively inhibited and eradicated biofilms formed by methicillin-sensitive and methicillin-resistant *S. aureus* strains, resulting in significant reductions in both biomass and viability [21]. Similarly, Millezi et al. (2019) demonstrated that Eug-based solutions, whether isolated or present in cinnamon essential oil, markedly reduced biofilm formation and viable cell numbers in *E. coli* and *S. aureus* [34]. Previous studies involving phenolic compounds suggest that eugenol may interfere with bacterial membranes and cell-to-cell communication (*quorum sensing*), which could contribute to its antibiofilm activity [4, 22].

Eug’s antimicrobial activity was assessed using conventional culture-based methods, including MIC and CFU enumeration. These approaches may overlook VBNC cells, an underestimated and controversial microbial survival strategy characterized by loss of culturability while retaining viability [15]. Therefore, reductions in CFU observed should be interpreted as reductions in culturable bacterial populations rather than complete eradication. Complementary culture-independent viability approaches would be required to determine whether VBNC populations persist following Eug exposure.

The morphological alterations observed in SEM images further support that Eug disrupted preformed biofilms in representative Gram-negative and Gram-positive bacteria, impairing their structural integrity and adherence capacity. These outcomes are consistent with prior studies demonstrating Eug-based essential oils from *Ocimum* spp. compromise bacterial membrane integrity, leading to cell leakage and collapse of biofilm structure [24, 35]. Thus, structural alterations observed in SEM analyses, together with antibiofilm findings, suggest that Eug interferes with biofilm organization and bacterial adherence. However, these images should be interpreted qualitatively, since the biofilms were formed on glass coverslips, which may differ in structure and complexity from those formed on polystyrene. Further molecular and biochemical studies are needed to clarify the mechanisms underlying these effects.

The Eug combination with Cip and Oxa showed only additive interactions (see Table 3). Although this requires further experimental validation, additive interactions could permit reductions in antibiotic doses or provide decreased toxicity or selective pressure, as suggested by Malczak [36]. Similar effects have been reported in studies combining Eug with colistin, cefotaxime and Cip, or beta-lactams, reinforcing the idea that phytochemicals can serve as antibiotic additives [37–39]. Kong et al. suggest that Eug may increase membrane permeability, facilitate antibiotic entry and enhancing intracellular targeting [40]. The additive effects detected may support future investigations exploring formulations or combinatorial strategies involving Eug and conventional antibiotics. Therefore, additional cytotoxicity and selectivity assays in mammalian cells are needed to determine Eug’s safety profile and therapeutic window.

## Conclusion

Eug exhibited in vitro antibacterial and antibiofilm activity against representative Gram-positive and Gram-negative bacteria. The compound also affected bacterial growth kinetics and promoted biofilm disruption, as shown by reduced biomass, fewer culturable cells, and SEM analyses. In addition, additive interactions were observed between Eug and conventional antibiotics against resistant isolates. Overall, this study provides additional experimental evidence supporting Eug as a natural compound of microbiological interest, particularly regarding its activity against mature biofilms and its potential use in combinatorial antimicrobial strategies.

## Data Availability

No datasets were generated or analysed during the current study.
